# Peroxisome proliferator activator receptor gamma coactivator-1alpha (PGC-1α) improves motor performance and survival in a mouse model of amyotrophic lateral sclerosis

**DOI:** 10.1186/1750-1326-6-51

**Published:** 2011-07-19

**Authors:** Wei Zhao, Merina Varghese, Shrishailam Yemul, Yong Pan, Alice Cheng, Paul Marano, Sadiq Hassan, Prashant Vempati, Fei Chen, Xianjuan Qian, Giulio M Pasinetti

**Affiliations:** 1 Department of Neurology, Mount Sinai School of Medicine, New York, NY 10029, USA; 2 GRECC, James J. Peters Veterans Affairs Medical Center, Bronx, NY 10468, USA

## Abstract

**Background:**

Amyotrophic lateral sclerosis (ALS) is a devastating neurodegenerative disease that affects spinal cord and cortical motor neurons. An increasing amount of evidence suggests that mitochondrial dysfunction contributes to motor neuron death in ALS. Peroxisome proliferator-activated receptor gamma co-activator-1α (PGC-1α) is a principal regulator of mitochondrial biogenesis and oxidative metabolism.

**Results:**

In this study, we examined whether PGC-1α plays a protective role in ALS by using a double transgenic mouse model where PGC-1α is over-expressed in an SOD1 transgenic mouse (TgSOD1-G93A/PGC-1α). Our results indicate that PGC-1α significantly improves motor function and survival of SOD1-G93A mice. The behavioral improvements were accompanied by reduced blood glucose level and by protection of motor neuron loss, restoration of mitochondrial electron transport chain activities and inhibition of stress signaling in the spinal cord.

**Conclusion:**

Our results demonstrate that PGC-1α plays a beneficial role in a mouse model of ALS, suggesting that PGC-1α may be a potential therapeutic target for ALS therapy.

## Background

Amyotrophic lateral sclerosis (ALS), or Lou Gehrig's disease, is one of the most common adult-onset neurodegenerative diseases. ALS results in the progressive loss of upper and lower motor neurons and gradual muscle weakening, which ultimately will lead to paralysis and death. No apparent genetic links have been found in the majority of the ALS patients, but the disease was inherited in the remaining cases (about 10%) [1]. The first ALS gene identified was the copper-zinc superoxide dismutase (SOD1), and it is the most extensively studied gene. SOD1 accounts for about 20% of familial ALS cases [2]. Mutations cause SOD1 to undergo toxic misfolding and aggregation, possibly causing a heightened presence of reactive oxygen species. Among more than 90 mutations on the SOD1 gene that have been associated with ALS through various studies, the mutation of glycine 93 to alanine (G93A) has been particularly well-studied [3,4]. It has been used to create the popular SOD1-G93A transgenic mouse model of ALS [5].

Many mechanisms are involved in the pathology of ALS, including glutamate toxicity, oxidative stress, defective axonal transport, glia cell pathology and mitochondrial dysfunction. The mitochondrion is a vital organelle that performs multiple functions in aerobic cells. It is the major site of ATP production, maintaining calcium homeostasis, participating in calcium signaling, and regulating intrinsic apoptosis. Therefore, mitochondrial malfunction presents multiple effects on the cell, especially neurons with an elevated susceptibility to aging and stress. Mitochondrial pathology is a key player among working hypotheses in the study of ALS [6-8]. Altered mitochondrial electron transport chain (ETC) enzyme activities have been observed in ALS patients and ALS mouse models [4,9-12]. Treatment with creatine, which could enhance mitochondrial activity, was found to improve motor performance and survival time in SOD1-G93A mice [13].

The transcriptional coactivator peroxisome proliferator-activated receptor gamma co-activator-1α (PPARGC1A or PGC-1α) is a master regulator of mitochondrial biogenesis and oxidative metabolism [14]. In PGC-1α knockout mice, expression of genes that are responsible for mitochondrial respiration is markedly dulled and mitochondrial enzymatic activities are also decreased [15].

In this study, we crossed PGC-1α transgenic animal with SOD1-G93A transgenic animal to test the potential effect of PGC-1α in this mouse model of ALS.

## Results

### Characterization of PGC-1α transgenic animal

**Figure 1:**
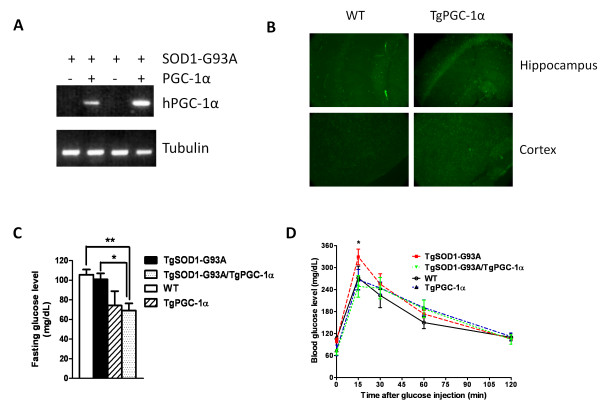
**PGC-1α expression and blood glucose level in transgenic animals**. (A) Expression of human PGC-1α transcript in the spinal cord; (B) Overexpression of PGC-1α in the brain of PGC-1α transgenic animal; (C) Base line blood glucose level in WT, PGC-1α, SOD1-G93A and SOD1-G93A/PGC-1α animals (*p < 0.05); (D) Glucose tolerance test of WT, PGC-1α, SOD1-G93A and SOD1-G93A/PGC-1α animals.

Since the PGC-1α gene inserted is on a rat neuron-specific enolase (NSE) promoter, we first examined the expression of inserted human PGC-1α in the mouse spinal cord. As expected, we only found human PGC-1α expression in the spinal cord of PGC-1α single transgenic and SOD1-G93A/PGC-1α double transgenic animals (Figure 1A). Then we looked at the expression level of PGC-1α in the brain. A significant overexpression of PGC-1α was observed in the hippocampus and cortex of PGC-1α transgenic mice (Figure 1B). We also examined SOD activity in these animals. A higher SOD enzymatic activity was observed in SOD1-G93A transgenic animals as previously described [16], but not in other experimental groups (data not shown).

### PGC-1α Reduced Blood Glucose Level in SOD1-G93A Mice

Impaired glucose tolerance has been reported in ALS patients [17]. To see whether presence of PGC-1α could have any beneficial effect in the glucose level, we performed a glucose tolerance test in the WT, PGC-1α, SOD1-G93A and SOD1-G93A/PGC-1α transgenic animals. We first compared the fasting blood glucose levels between the four groups (Figure 1C) and found that SOD1-G93A/PGC-1α double transgenic animals had a significantly lower baseline glucose reading than the SOD1-G93A animals (100.8 ± 6.111 vs. 69.00 ± 7.382 mg/dL, N = 5, p = 0.0106). We also recorded their glucose levels at 15, 30, 60, and 120 minutes after glucose injection (2 mg glucose per g body weight). Although two way ANOVA analysis did not reveal a major difference between the SOD1-G93A single transgenic animals and the SOD1-G93A/PGC-1α double transgenic animals, (Figure 1D), a significantly lower peak glucose level (at 15 min) was observed in the SOD1-G93A/PGC-1α double transgenic animals (Bonferroni post-test, p < 0.05).

### PGC-1α Improved Motor Performance and Survival in SOD1-G93A Mice

**Figure 2:**
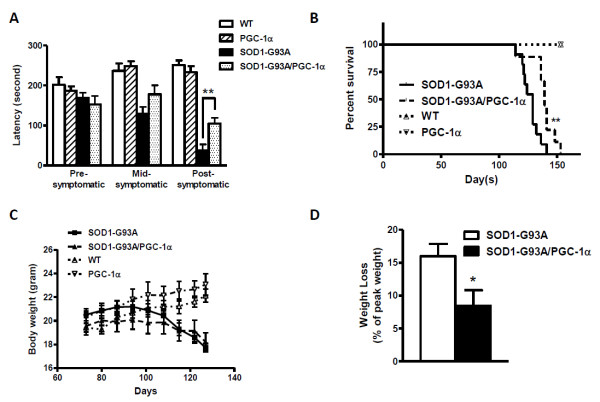
**PGC-1α improved motor performance and survival in SOD1 mutant mice**. (A) Motor performance as measured by rotarod test in WT, PGC-1α, SOD1-G93A and SOD1-G93A/PGC-1α animals (**p < 0.01); (B) Survival analysis of WT, PGC-1α, SOD1-G93A and SOD1-G93A/PGC-1α animals (**p < 0.01 by Mantel-Cox test); (C) Average body weight of WT, PGC-1α, SOD1-G93A and SOD1-G93A/PGC-1α animals; (D) Average weight loss (percentage of peak weight) of SOD1-G93A and SOD1-G93A/PGC-1α animals (*p < 0.05).

Important behavioral and physiological characteristics of SOD1-G93A transgenic mice include impaired motor performance, weight loss and reduced survival as compared to the wild-type. We assessed motor performance of the single and double transgenic animals using an accelerating rotarod apparatus for mice. At pre-symptomatic/mid-symptomatic stages, there was no apparent difference in the motor function between SOD1-G93A and SOD1-G93A/PGC-1α double transgenic animals. Remarkable improvement in the motor function was observed in the double transgenic as compared to the ALS mice at post-symptomatic stage (week 17) (latency of 104.3 ± 14.42 vs. 37.13 ± 15.80 s, N = 8, p = 0.0078) (Figure 2A).

We also assessed the survival of the experimental animals. SOD1-G93A/PGC-1α double transgenic animals have a median survival of 139 days, which is significantly longer than SOD1-G93A animals with a median survival of 129 days (Figure 2B) as determined by the Mantel-Cox test (p = 0.0064).

The body weight of each mouse was monitored weekly during the study period (Figure 2C). We compared the peak weight at pre-symptomatic stage (day 87) with the weight at their post-symptomatic stage (day 127), and found that SOD1-G93A/PGC-1α double transgenic animals had a significantly less weight loss (percentage of peak weight) than their SOD1-G93A littermates (two-way t-test, p = 0.0214) (Figure 2D).

### PGC-1α Protected Against Motor Neuron Death in SOD1-G93A Mice

**Figure 3:**
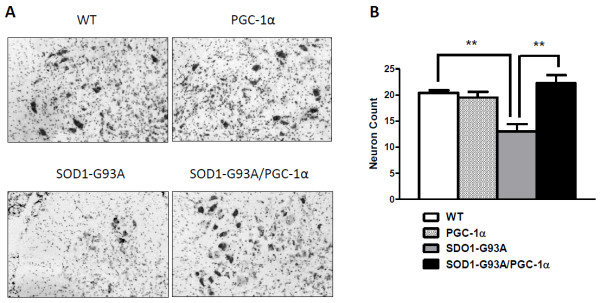
**Nissl-stained motor neuron count in the lumbar spinal cord**. The effect of the PGC-1α on neuron numbers in SOD1-G93A transgenic mice at post-symptomatic stage (day 110) was examined following Nissl-staining. (A) Photomicrographs of representative Nissl-stained sections through the ventral horns of the lumbar spinal cord from wild-type (WT), SOD1-G93A, PGC-1α and SOD1-G93A/PGC-1α double transgenic mice. (B) Motor neuron counts in lumbar spinal cord (data = Mean ± SE, n = 5, **p < 0.05).

To determine whether PGC-1α can protect against the motor neuron loss that accompanies the clinical symptoms of ALS, we counted the number of motor neurons in the lumbar spinal cord in age and gender matched WT, SOD1-G93A, PGC-1α and SOD1-G93A/PGC-1α mice at post-symptomatic stage (day 110) (Figure 3A). Wild type animals had a mean of 20.40 ± 0.5099 (N = 5) motor neurons per spinal cord section. As expected, motor neuron counts in SOD1-G93A mice were significantly lower compared to WT (p < 0.01). In SOD1-G93A/PGC-1α double transgenic mice, there were significantly more motor neurons in the ventral horn compared to those in the SOD1-G93A mice (22.25 ± 1.548 vs. 13.00 ± 1.414, p = 0.003) (Figure 3B).

### PGC-1α Restored Mitochondrial Electron Transport Chain Activities in the Spinal Cord

**Figure 4:**
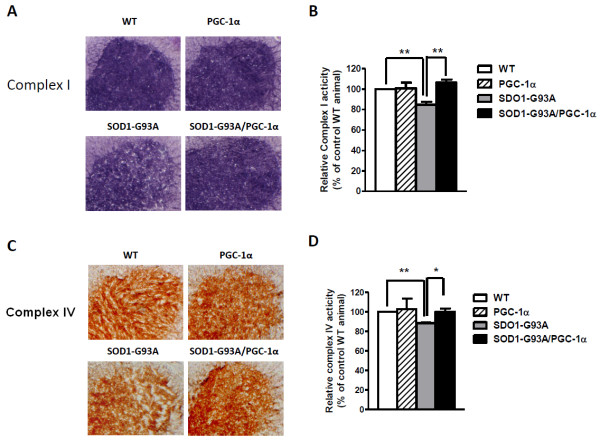
**Detection and quantification of mitochondrial electron transport chain complex activities in the ventral horn of lumbar spinal cord**. (A) Representative images of complex I activity in the four experimental groups; (B) Quantification of complex I activity (*p < 0.05); (C) Representative images of complex IV activity in the four experimental groups; (D) Quantification of complex IV activity (*p < 0.05).

To evaluate the effect of PGC-1α in mitochondrial ETC activities, we isolated the spinal cords of age, gender-matched WT, SOD1-G93A, PGC-1α and SOD1-G93A/PGC-1α animals. In situ histochemical assays were performed in the lumbar spinal cord sections as previously described [13,18]. We detected a decrease of complex activities in the ventral horn of lumbar spinal cord in mutant SOD1-G93A animals as compared to their wild-type littermates. Notably, SOD1-G93A/PGC-1α double transgenic animals showed a similar level of complex I (Figure 4A, B) and complex IV activities (Figure 4C, D) as wild-type animals, suggesting that the presence of PGC-1α attenuated the mitochondrial ETC transport defect in mutant ALS animals. No significant improvement of complex II activity was observed in SOD1-G93A/PGC-1α double transgenic animals when compared to SOD1-G93A animals (data not shown).

### PGC-1α Decreased Phosphorylation of JNK and p38 MAPK in SOD1-G93A Mice

**Figure 5:**
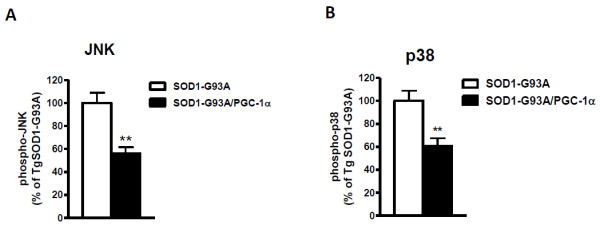
**PGC-1α inhibited phosphorylation of JNK and p38 MAPK**. Bead-based multiplex luminex assay was performed to examine the phosphorylation of (A) JNK and (B) p38 MAPK. Mean fluorescence intensities were recorded and data were plotted as percentage of SOD1-G93A animals (*p < 0.01).

Since hyper-phosphorylation of stress-activated kinases JNK (c-jun N-terminal kinase) and p38 MAPK has been demonstrated in SOD1-G93A mice [13,19,20], we used a bead-based multiplex luminex assay to examine the phosphorylation of JNK (Thr183/Tyr185) and p38 (Thr180/Tyr182). In spinal cord lysates of SOD1-G93A/PGC-1α double transgenic animals, significantly less amount of phospho-JNK (Figure 5A, p = 0.0025) or phospho-p38 (Figure 5B, p = 0.0064) was detected as compared to the single transgenic mice.

## Discussion

Our study demonstrated that SOD1-G93A/PGC-1α double transgenic animals showed significant improvements in the clinical signs of ALS. The improvement of motor performance in SOD1-G93A/PGC-1α double transgenic animals was accompanied by a significant preservation of motor neurons and mitochondrial electron transport chain activities in the ventral horn of the lumbar spinal cord. Additionally, SOD1-G93A/PGC-1α double transgenic animals showed remarkably less weight loss at their end stages and had a notably longer life span as compared to their SOD1-G93A littermates.

Transport of materials (protein and organelles) between the cell body and neuron processes is essential to signal transduction and neuronal survival. Disruption of slow axonal transport of the cytoskeleton is one of the earliest pathological events in mutant SOD1 mice [21]. Fast axonal transport is responsible for transporting membrane-bound organelles, such as mitochondria, to maintain axonal and synaptic function. While it remains unclear how impairment of axonal transport causes motor neuron dysfunction and degeneration, some transport cargos such as neurotrophic factors, signaling molecules and mitochondria are obvious targets. Increased stress signaling (p-JNK, caspase-8 and p75NTR) was recently found in SOD1-G93A mice [19]. The p38 stress-activated kinase is activated and phosphorylates neurofilaments in SOD1-G93A mice [20,22]. p38 is also involved in regulating fast axonal transport including transport of mitochondria [23,24]. Our data showed a significant decrease of phosphorylation of JNK at Thr183/Tyr185 and p38 MAPK at Thr180/Tyr182, suggesting that the neuroprotective effect of PGC-1α in SOD1-G93A mice might partially work through inhibiting the stress-activated signaling pathway and/or regulating mitochondria transport.

A growing amount of evidence identifies PGC-1α as a potential therapeutic target in neurodegenerative diseases, including Alzheimer's disease (AD), Parkinson's disease (PD), and Huntington's disease (HD). Genome-wide expression studies revealed that PGC-1α might be a therapeutic target for early intervention in PD [25]. Previous studies from our lab showed that PGC-1α expression decreased in AD patient brain as a function of dementia [26]. PGC-1α is implicated to play a key role in controlling energy metabolism in the early stages of HD pathogenesis [27,28]. To date there is no direct evidence that PGC-1α plays a role in ALS pathogenesis. However some circumstantial evidence suggests that this represents a line of investigation worth pursuing. For instance, thiazolidinediones (TZDs) such as rosiglitazone and pioglitazone, which induce PGC-1α expression and activate the PPARγ pathway have been shown to be beneficial in the SOD1 transgenic mouse model of ALS by two independent research groups [29,30]. Our *in vivo *evidence, for the first time demonstrates the neuro-protective role of PGC-1α in an ALS mouse model, suggesting that PGC-1α might be a potential therapeutic target for early intervention in ALS.

## Methods

### Experimental animals

Rat neuron-specific enolase (NSE) promoter plasmid containing hPGC-1α was constructed by inserting 3.1 kb cDNA fragment with entire coding region of hPGC-1α (NM_013261.2, OriGene Technologies, Inc. Rockville, MD) in Not I site of the plasmid vector. A cassette of ~8 kb SalI fragment containing NSE promoter and hPGC-1α was gel purified and microinjected into one-cell mouse egg (C57BL6 × SJL)as described previously [31,32]. TgPGC-1α founders were identified by PCR-based genotyping.

Male TgSOD1-G93A mutant transgenic mice (C57BL6 × SJL) were purchased from the Jackson Laboratory and bred with female PGC-1α transgenic mice in our transgenic mouse facility to generate TgSOD1-G93A/TgPGC-1α double transgenic mice and their TgSOD1-G93A, TgPGC-1α or wild-type littermates. Mice were housed on a 12-hour-light, 12-hour-dark cycle and allowed ad libitum access to food. Mice were weighed weekly starting from 8 weeks of age. The survival study endpoint was defined as meeting any one of the following conditions: no spontaneous breathing or movement for 60 seconds with no response to pain; the animal is unable to roll over the normal position within 10 seconds following a push over; or complete hind limb paralysis. The Institutional Animal Care Committee of Mount Sinai School of Medicine reviewed and approved all experimental protocols used in this study.

### Immunostaining

The brains of 5 months old Wild type and Tg2576 mice were dissected, cut into two hemispheres and fixed in 4% Paraformadehyde. After extensive wash with PBS, equilibrated with 30% sucrose and embedded in Tissue Freezing Medium (Triangle Biomedical Sciences). The frozen brains were transversely sectioned (14 uM). The tissue sections were incubated with PGC-1α antibody (Santa Cruz Technology, H-300, 1:500 dilution) overnight at 4°C. The sections were then washed and incubated with FITC-conjugated secondary antibody (1:250 dilution) for 1 hour. Following several washes with PBS, images were acquired under fluorescence microscope.

### Motor Function Assessment

TgSOD1-G93A/TgPGC-1α mice and TgSOD1-G93A mice were tested on an accelerating rotarod (7650 Ugo Basile Biological Research Apparatus, Comerio, Italy) as previously described. In brief, mice are placed onto a grooved cylinder (facing away from the experimenter) rotating at a predetermined speed that incrementally increases to a maximal rotation at 300s; the time maintained on the rod by each mouse (latency) is then recorded (300 s max). A diminishing latency indicates declining performance and at values of 0 s is suggestive of severe muscular weakness and impaired coordination. Mice were tested beginning at 80 days of age weekly until they could no longer perform the test. Before testing, mice underwent a 1 week training period wherein they were introduced to the apparatus and handled by the experimenter daily. Testing was conducted during the last 4 h of the day portion of the light cycle in an environment with minimal stimuli such as noise, movement, or changes in light or temperature.

### Glucose Tolerance Test

Mice were fasted overnight in clean cages with free access to water in new clean bottles. The next morning each mouse was weighed, and a baseline fasted blood glucose measurement was taken by applying tail blood to a Contour Blood Glucose Monitoring System (Bayer). Each mouse was injected intraperitonealy with a filter-sterilized solution of 20% (w/v) D-glucose, with the size of the bolus determined by animal weight (2 mg glucose/g body weight). Blood glucose measurements were taken as described above for each animal at 15, 30, 60 and 120 minutes. The data were plotted as blood glucose concentration (mg/dL) over time (minutes).

### Luminex Assay

A Bead-based multiplex luminex assay was performed using MILLIPLEX MAP 8-Plex Multi-Pathway Signaling Kit, Phosphoprotein (Millipore, Billerica, MA) following the manufacturer's protocols. Briefly, the spinal cords were homogenized in ice-cold Milliplex lysis buffer with protease inhibitors and then centrifuged at 12,000 rpm for 10 minutes at 4°C. Protein concentration was measured using the Bradford method. 25 μg protein of each sample was used for analysis.

### Preparation of Mouse Spinal Cord Sections

Age and gender-matched wild type, TgSOD1-G93A, TgPGC-1α and TgSOD1-G93A/TgPGC-1α double transgenic mice were euthanized by ketamine and their spinal cords were dissected out. The lumbar region was separated and rapidly frozen under 2-methylbutane on dry ice. The samples were stored at -80°C until sectioned. For sectioning, samples were embedded in OCT compound and transverse sections were cut at -20°C using a Leica CM3050 cryostat. The sections were collected on positively-charged glass slides (Superfrost Plus, Fisher Scientific) and stored at -80°C till use.

### Histology

Ten serial sections (25 μm thick) were cut 350 μm apart through the lumbar (L3-L5) spinal cord of each animal (n = 5). The sections were mounted onto Superfrost Plus slides and Nissl staining was performed using cresyl violet as previously described [33]. Large (>25 μm), Nissl stained neurons were counted within the ventral horns under a light microscope at a magnification of 200.

### Histochemical Reaction, Imaging and Analysis

8 μm thick sections of the lumbar spinal cord were air dried for 30 min and used for activity staining of mitochondrial complexes [1,18]. All the staining reactions were carried out at room temperature in the dark. To quantify complex I activity, the sections were incubated in 0.1 M Tris-HCl (pH 7.4), 0.14 mM NADH, 1 mg/ml nitroblue tetrazolium, 2 μg/ml antimycin, 84 mM malonate and 2 mM potassium cyanide. For complex II histochemistry, the enzyme was activated [34,35] by a 10 min incubation in 0.05 M potassium phosphate buffer (pH 7.4), followed by the addition of the reaction mix consisting of 4.5 mM EDTA, 2 mg/ml nitroblue tetrazolium, 50 mM succinate, 0.2 mM phenazonium methosulfate, 2 μg/ml antimycin, 60 μM rotenone and 2 mM potassium cyanide in the same buffer. The complex IV reaction required 75 mg/ml sucrose, 1 mg/ml diaminobenzidine HCl, 24 U/ml catalase, 1 mg/ml cytochrome c, 2 μg/ml antimycin, 60 μM rotenone and 84 mM malonate in 0.05 M potassium phosphate buffer (pH 7.4). The negative controls contained 60 μM rotenone, 84 mM malonate or 2 mM potassium cyanide to specifically inhibit complexes I, II or IV respectively. After incubations of 20 min for the complex I and IV reactions or 10 min for the complex II reaction, the sections were washed twice in phosphate buffered saline, once in distilled water and then mounted in glycerin jelly.

Stained sections were visualized under an Olympus MVX10 Macroview stereomicroscope controlled by MicroSuite Five Biological Suite software and photographed using the attached Hamamatsu C8484 monochrome camera. Images were taken with particular care to use uniform gray scales and below the level of saturation. Optical intensities of the ventral horn area on these sections were quantified using ImageJ software (NIH). Optical intensities were converted to optical densities (OD) by the formula: OD = log10 (Ibk/Im), where Ibk is background intensity and Im is measured intensities from different regions of the sections.

## Abbreviations

ALS: amyotrophic lateral sclerosis; PPARGC1A or PGC-1α: peroxisome proliferator-activated receptor gamma co-activator-1α; SOD: copper-zinc superoxide dismutase; ETC: electron transport chain; NSE: neuron-specific enolase; JNK: c-jun N-terminal kinase; AD: Alzheimer's disease; PD: Parkinson's disease; HD: Huntington's disease;

## Competing interests

The authors declare that they have no competing interests.

## Authors' contributions

WZ: conceptualized the project, designed and carried out the experiments, analyzed data and drafted the manuscript; MV: carried out histochemical assays and participated in manuscript revision; SY: carried out vector construction; YP: carried out immunostaining; PV and FC: participated in animal motor function assessment; AC and XQ: contributed to animal care, genotyping and dissection; PM and SH: participated in histology and histochemical assays; GMP: involved in project conception and experimental design, data interpretation, manuscript preparation and final approval. All authors have read and approved this manuscript.
